# Mobile alcohol-specific inhibition training in adolescents and young adults with alcohol use disorder: study protocol for a randomized controlled feasibility trial

**DOI:** 10.3389/fpsyg.2026.1684231

**Published:** 2026-04-22

**Authors:** Kirstin Schuerch, Thomas Koenig, Alessandra Guarriello, Leila Soravia, Thomas Berger, Kristina Adorjan, Franz Moggi, Maria Stein

**Affiliations:** 1 University Hospital of Psychiatry and Psychotherapy, Translational Research Center, University of Bern, Bern, Switzerland; 2 Clinic Suedhang, Center for Treatment of Addictive Disorders, Kirchlindach, Switzerland; 3 Department of Clinical Psychology and Psychotherapy, Institute of Psychology, University of Bern, Bern, Switzerland

**Keywords:** alcohol use disorder, adolescence and young adults, inhibition training, cognitive bias modification, feasibility trial, pilot study, mobile health

## Abstract

Alcohol misuse is a major global risk factor for morbidity and mortality among adolescents and young adults. During adolescence and young adulthood, alcohol can disrupt brain maturation, alter brain connectivity, and persistently weaken self-regulation. The limited effectiveness of current evidence-based treatments underscores the need for adjunctive interventions. Neuroscientific models suggest that an imbalance between the appetitive system and executive control processes contributes to alcohol misuse. Alcohol-specific inhibition training (Alc-IT) addresses this imbalance and has shown first indications of beneficial effects in inpatient settings. However, app-based delivery and its use in adolescents and young adults are novel and have not yet been systematically investigated for feasibility, preliminary effectiveness, and neurophysiological effects. This multicenter, double-blind, randomized controlled feasibility trial aims to assess the implementation of app-based Alc-IT as an add-on to outpatient treatment for alcohol use disorder (AUD). Up to 210 participants (14–27 years) will be randomized to Alc-IT or an active control condition. Both groups complete six training sessions over a two-week period and six booster sessions one month later. Feasibility outcomes include recruitment and completion rates. Changes in drinking behavior will be assessed at one-month and three-month follow-up using the Timeline Follow-Back method. In a sub-study with up to 60 participants, neurophysiological correlates of inhibitory control (N2 and P3 event-related potentials) will be measured. EEG will be recorded during a modified Go/NoGo task before and after training. This study will inform the feasibility, preliminary effectiveness, and potential neurophysiological mechanisms of app-based Alc-IT for young people with AUD. The findings will guide the design of a subsequent fully powered randomized controlled trial.

**Clinical trial registration:**

ClinicalTrials.gov, ID: NCT07071779; SNCPT, ID: SNCTP000006292.

## 1 Background

Adolescence and young adulthood represent developmental periods characterized by increased exploration and risk-taking behavior including alcohol misuse (Casey et al., 2008; Steinberg, 2008). In adolescents and young adults, alcohol misuse is a leading global risk factor for death and adverse effects, significantly impacting individuals and their social environments (Nutt et al., 2010). Early initiation of alcohol use has been associated with a range of adverse outcomes, including poorer cognitive functions, mental health problems, and increased vulnerability to developing substance use disorders (Danpanichkul et al., 2025; Spear, 2018).

Although evidence-based treatments for alcohol use disorder (AUD) exist, their effectiveness is limited by, amongst others, insufficient access and low motivational appeal. As a result, many patients remain untreated or without optimal care (Ritter et al., 2019). Even among those who do receive treatment, non-response and high relapse rates are common (Bottlender and Soyka, 2005; Ray et al., 2019). One way to narrow the treatment gap is to bring optimized interventions closer to patients, which can be achieved through web-based interventions delivered on patients’ mobile phones. Such low-threshold approaches increase the capacity to reach a larger proportion of patients (Smit et al., 2011) and may be highly appealing to younger, “digital native” patients.

Neuroscientific models of AUD and other addictive disorders highlight an imbalance between sensitized appetitive system and weakened inhibitory control (Volkow and Baler, 2014; Wiers et al., 2007). Patients with AUD often exhibit impaired inhibitory control, particularly when exposed to alcohol-related cues (Smith et al., 2014; Stein et al., 2018). Reduced inhibitory control is more pronounced in adolescence due to the ongoing maturation of the prefrontal cortex (Wiers et al., 2007). Conventional psychotherapeutic approaches do not directly address these automatic, cue-triggered processes. However, effective modification of the underlying neuronal circuits should include repetitive, implicit learning to counteract automatized appetitive processes (Lindenmeyer, 2010; Stacy and Wiers, 2010; Wiers et al., 2007).

Translating these findings into a therapeutic application, alcohol-specific inhibition training (Alc-IT) directly targets these crucial processes. Alc-IT refers to computerized interventions that aim to strengthen inhibitory control in response to alcohol-related cues. To date, most studies have implemented a modified Go/NoGo paradigm, in which alcohol-related stimuli are consistently paired with a stopping response. The theoretical rationale is to counteract automatic approach tendencies toward alcohol by training cue-specific inhibitory control (Batschelet et al., 2020). After a series of preclinical proof-of-concept studies (see Batschelet et al., 2020 for a review), Alc-IT has led to promising results in a first multicentric, double-blind, randomized-controlled clinical trial (RCT). In this study, it reduced drinking in a sample of inpatients with AUD (Stein et al., 2023; but see Schenkel et al., 2023). Alc-IT was originally intended to modify neurophysiological alterations that had been linked to poor inhibition in AUD. Therefore, it is of high interest to monitor whether Alc-IT modifies these crucial processes (i.e., whether Alc-IT displays the expected neurophysiological effects).

At the neurophysiological level, inhibitory control is often studied with a Go/NoGo task. Two event-related potentials (ERP), the N2 and P3 components, reliably appear during NoGo trials (Campanella et al., 2019b; Zhang et al., 2021). The NoGo N2 is a frontocentral negative peak around 200–400 ms after stimulus onset, reflecting conflict monitoring (Donkers and van Boxtel, 2004; Huster et al., 2013). The NoGo P3, a frontocentral positive peak around 300–600 ms after stimulus onset, reflects actual inhibitory control (Fallgatter and Strik, 1999; Smith et al., 2008). Studies in healthy participants show that inhibition training can alter the N2 (Benikos et al., 2013; Schapkin et al., 2007; Schroder et al., 2020). Findings comparing patients with AUD and healthy controls on NoGo N2 and P3 have been inconsistent (Colrain et al., 2011; Dubuson et al., 2023; Kamarajan et al., 2005; Pandey et al., 2012). In contrast, studies focusing on alcohol-related Go/NoGo tasks revealed clinically relevant results: Alcohol-related NoGo N2 amplitudes increase with craving and reduced NoGo P3 predicts relapse (Batschelet et al., 2021; Campanella et al., 2019a; Petit et al., 2014; Stein et al., 2018).

However, the application of Alc-IT in adolescent and young adult populations, especially in mobile formats, remains unexplored. Computerized interventions such as Alc-IT can be a cost-effective add-on to traditional relapse prevention strategies. To fully unlock the potential of these interventions, they need to be both appealing and easily accessible for patients. Furthermore, they should be integrated into comprehensive, evidence-based programs, and implemented in a way that optimally targets the underlying neuronal processes (Holmes et al., 2018; Wiers et al., 2018). Delivering Alc-IT via a mobile app is one way to expand its availability beyond the residential treatment settings. This approach has worked for similar forms of cognitive bias modification, such as approach bias modification (Laurens et al., 2020; Manning et al., 2021; Prior et al., 2021). The present project administers for the first time a newly developed, app-based version of Alc-IT in addition to outpatient treatment as usual in a sample of adolescents and young adults with AUD. The study has three objectives:

### 1.1 Primary objective

#### 1.1.1 Objective 1: assessing feasibility of Alc-IT

Feasibility will be assessed by recruitment and completion rates. The hypothesis regarding feasibility, albeit not requesting inferential statistics, are formulated according to the relevant guidelines (Eldridge et al., 2016). The expectations are based on (a) the mean percentages reported in earlier studies with similar interventions (Manning et al., 2021; Stein et al., 2023) and (b) the admission rates at the participating treatment centers (see footnote 1):

Hypothesis for objective 1

*H1*: We expect promising feasibility as indicated by successful recruitment of up to 210 patients, ≥ 62% completion rates of the core training phase, and ≥43% completion of the booster trainings phase.

Additionally, we will document adherence (number of completed training sessions), occurrence of adverse events, and whether participants receive Alc-IT as intended (i.e., as an add-on to standard care).

### 1.2 Secondary objectives

#### 1.2.1 Objective 2: gain preliminary findings regarding the effectiveness of Alc-IT on a drinking outcome

The second objective is to obtain preliminary evidence regarding the effects of Alc-IT on drinking behavior as an add-on to standard care. Unlike the original Alc-IT assessed in inpatient settings with supervised sessions (Stein et al., 2023), this app-based version allows independent use in everyday contexts. This study thus aims to gain preliminary insight into the effectiveness on drinking behavior of Alc-IT as an add-on to standard care in adolescents and young adults with AUD. As the international CONSORT guidelines state that formal hypothesis testing for effectiveness is not recommended for pilot and feasibility trials (Eldridge et al., 2016), no hypotheses (H2) for this secondary objective are formulated. However, the research aim here is to describe Alc-IT’s effects on drinking behavior, as indicated by change in percentage of days abstinent and change in heavy drinking days (both assessed with the timeline follow-back method, Sobell and Sobell, 1992) in terms of effect sizes and measures of uncertainty.

#### 1.2.2 Objective 3: EEG sub-study – investigate the neurophysiological effects of Alc-IT within a subsample

The third objective is to investigate whether app-based Alc-IT can modify the neurophysiological anchorage of alcohol-specific inhibition. Such an effect would indicate that the more flexible administrable version of Alc-IT is also effective at normalizing crucial neurophysiological alterations. Building on the fact that online interventions are typically characterized by a higher variation in sessions completed (Manning et al., 2021), we will also investigate whether the neurophysiological effects vary with the number of sessions completed. This analysis will yield valuable insight regarding the optimal number of training sessions, for which evidence-based information is still lacking.

Hypothesis for objective 3:

*H3*: We expect that in patients receiving Alc-IT, the topographical difference between neutral and alcohol-related NoGo trials in the P3 component will be reduced from pre- to post-training to a larger extent than in the control group.

Furthermore, we exploratorily investigate whether baseline levels of these neurophysiological parameters, the personality trait impulsivity, or performance levels during training function as moderators of potential treatment effects.

## 2 Methods

### 2.1 Study design

The present study combines a multicentric, double-blind feasibility RCT with two parallel study arms to evaluate feasibility (Objective 1) and explore preliminary effectiveness (Objective 2) with an experimental neurophysiological proof-of-concept study assessing the neurophysiological effects of Alc-IT (Objective 3). Following the relevant CONSORT guidelines (Eldridge et al., 2016) and similar to recent feasibility RCTs in this domain (Manning et al., 2021; Manning et al., 2020; Prior et al., 2021), the present study will collect indicators for feasibility from patients and care providers (Objective 1), while also examining preliminary effectiveness by comparing drinking behavior change from pre- to post-treatment between the Alc-IT group and an active control group (Objective 2). The EEG sub-study is designed as an experimental neurophysiological study, which will assess neurophysiological effects of Alc-IT to delineate its working mechanisms as well as moderators of treatment effects on a neuronal level (Objective 3).

### 2.2 Study population

A total of up to 210 adolescents and young adults with AUD currently undergoing outpatient treatment or (online) counselling in one of 5 specialized treatment settings within Switzerland will be recruited.

#### 2.2.1 Eligibility criteria

Participants fulfilling all of the following inclusion criteria are eligible for the study; (1) age 14–27 years, (2) currently undergoing outpatient treatment or online counselling in one of participating specialized treatment centers, (3) a score of ≥ 8 in the Alcohol Use Disorder Identification Test (AUDIT; Babor et al., 2001), (4) a score of ≥ 2 in the Alcohol Use Disorder Screening (AUD-S; Knight et al., 2002), (5) sufficient German language skills, (6) owner of a smartphone with internet access. The presence of any one of the following exclusion criteria will lead to exclusion of the participant: (1) Other severe substance use (except nicotine and cannabis) determined by values ≥ 25 in the Drug Use Disorder Identification Test (DUDIT; Berman et al., 2005), (2) current medical conditions excluding participation.

Additional exclusion criteria for EEG sub-study are as follows: (1) Current medication affecting EEG (e.g., benzodiazepines), (2) other severe substance use (except nicotine) determined by values ≥ 25 in the DUDIT, (3) history of epilepsy, (4) cochlea implant.

### 2.3 Recruitment and screening

Participants will be recruited in collaboration with five specialized outpatient treatment or online counselling settings. Initial contact will be made by treatment providers or study team members. Individuals expressing interest will receive written and verbal information about the study. If they agree to participate, written informed consent will be obtained. In case of minors, informed consent of parents or legal representatives will also be obtained. After obtaining informed consent, participants will undergo formal screening to determine their eligibility.

**Figure 1:**
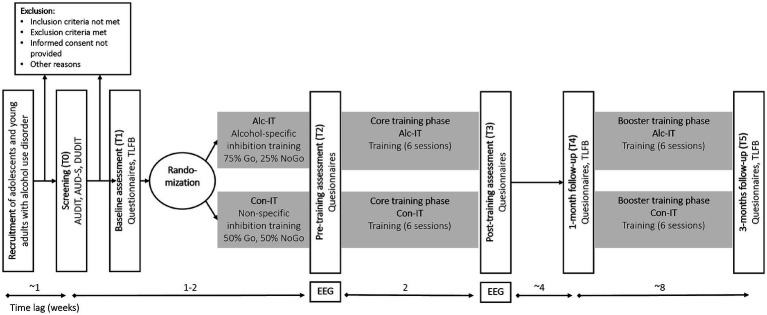
Study design and participant flow. T0 to T5 denote different assessment time points, ranging from screening to 3-month follow-up. Note that for the EEG sub-study, additional EEG measurements will be conducted at pre- (T2) and post-training (T3) in a subsample. See Table 1 for an overview of questionnaires and measures gathered at each assessment. AUDIT = Alcohol Use Disorder Identification Test (Babor et al., 2001), AUD-S = Alcohol Use Disorder Screening (Knight et al., 2002), DUDIT = Drug Use Disorder Identification Test (Berman et al., 2005), TLFB = Timeline Follow-Back (Sobell and Sobell, 1992; Sobell and Sobell, 1995), EEG = electroencephalography, Alc-IT = mobile alcohol-specific inhibition training, Con-IT = mobile non-specific inhibition training.

Due to additional time-consuming measurements in the EEG sub-study, a two-leveled recruitment process will be implemented: Participants in the main study will be asked if they are interested in participating in additional EEG measurements during the pre- (T2) and post-training assessment (T3; see Figure 1). If so, additional information for the EEG sub-study will be provided. If the participant agrees to participate, additional written informed consent and screening will be obtained from the participants.

**Table 1:** Schedule of enrolment, treatment and assessments according to SPIRIT.

Timepoint	Study period								
	Enrolment		Allocation	Post-allocation			Follow-up		
	T0	T1		T2	IT	T3	T4	IT	T5
Enrolment									
Eligibility screen	X								
Study information	X								
Informed consent	X		X						
Randomization									
Interventions									
Alcohol-specific inhibition training (Alc-IT)					X			X	
Control training (Con-IT)					X			X	
Assessments									
Feasibility Recruitment and completion rates, treatment adherence, and adverse events		X		X	X	X	X		X
Drinking behavior TLFB		X					X		X
Usability, user experience, and acceptability SUS, UEQ, Web-CLIC						X			
Open-text questions						X			X
Diagnostics AUDIT,	X						X		X
AUD-S,	X								
DUDIT	X						X		
AUD, psychopathology, and related measures HDL, Drinking goals		X					X		X
BSCL, DERS		X							
WHOQOL-BREF, SOCRATES, OCDS-G		X					X		
Craving, Motivation		X		X		X	X		X
Baseline measures (Socio-)demographics, I-8, ASRS-V1.1		X							
Neurophysiological effects (EEG sub-study) EEG assessment including BIS/BAS, GNG task				X		X			

ASRS-V1.1: adult ADHD self-report scale, including impulsivity subscale, AUDIT: Alcohol use disorders identification test; BIS/BAS: behavioral inhibition system/behavioral approach system scale; BSCL: brief symptom check list; Craving: likert scales measuring craving; DERS: difficulties in emotion regulation scale; GNG task: Go-NoGo task; HDL: health and daily living form, including measures on heavy drinking and days abstinent; I-8: scale for impulsive behavior; Motivation: visual analogue scale to measure motivation; OCDS-G: Obsessive compulsive drinking scale – German; SOCRATES: stages of change readiness and treatment eagerness scale; SUS: system usability scale; TLFB: timeline follow-back, from which measures of heavy drinking days and days abstinent will be derived; UEQ: user experience questionnaire; open-text questions concerning drawbacks and strength of the app, technical problems encountered, suggestions for amelioration, intention to continue using the app, place of using the app; Web-CLIC: facets of website content questionnaire; WHOQOL-BREF: WHO quality of life scale.

### 2.4 Randomization and blinding

After the baseline assessment (T1), participants are allocated to one of two study groups (see Figure 1) by a secure web-based system hosted at the University of Bern using a randomization list stratified by age, gender, and treatment center.

Participants, clinical staff and members of the study team will be blinded. An exception is made for those members of the study team, who administrate the app. However, they do not have direct contact with the patients. To ensure blinding, all study personnel, especially outcome assessors, will be instructed not to engage in discussions with patients concerning their assumptions regarding the allocated training version.

### 2.5 Sample size determination

Note that the present feasibility RCT is not designed to statistically test hypotheses regarding effectiveness outcomes but will rather yield preliminary findings into the effect size to be expected in a future full scale RCT (Eldridge et al., 2016; Thabane et al., 2016; Thabane and Lancaster, 2019). Sample size determination followed recommendations for sample sizes in pilot and feasibility trials that allow for an acceptable estimation of future effect sizes. These recommendations suggest that for small effects, sample sizes ranging from 25 to 75 per study arm are sufficient (Whitehead et al., 2016). Given the recruitment estimation in the five treatment centers^1^, we expect to collect complete data for drinking behavior outcome from 90 patients, which aligns with these recommendations.

For the EEG sub-study, a power analysis was conducted. Using the observed neurophysiological effect size of 0.255 in our prior study (Tschuemperlin et al., 2019) as an estimate and adapting power analyses performed with R to randomization statistics (Manly, 2007), the relevant effects can be detected with a power of 0.8 and alpha-level of 0.05 starting from a sample size of 55. Therefore, complete data will be collected from 60 patients in the EEG sub-study.

### 2.6 Procedure

**Figure 2:**
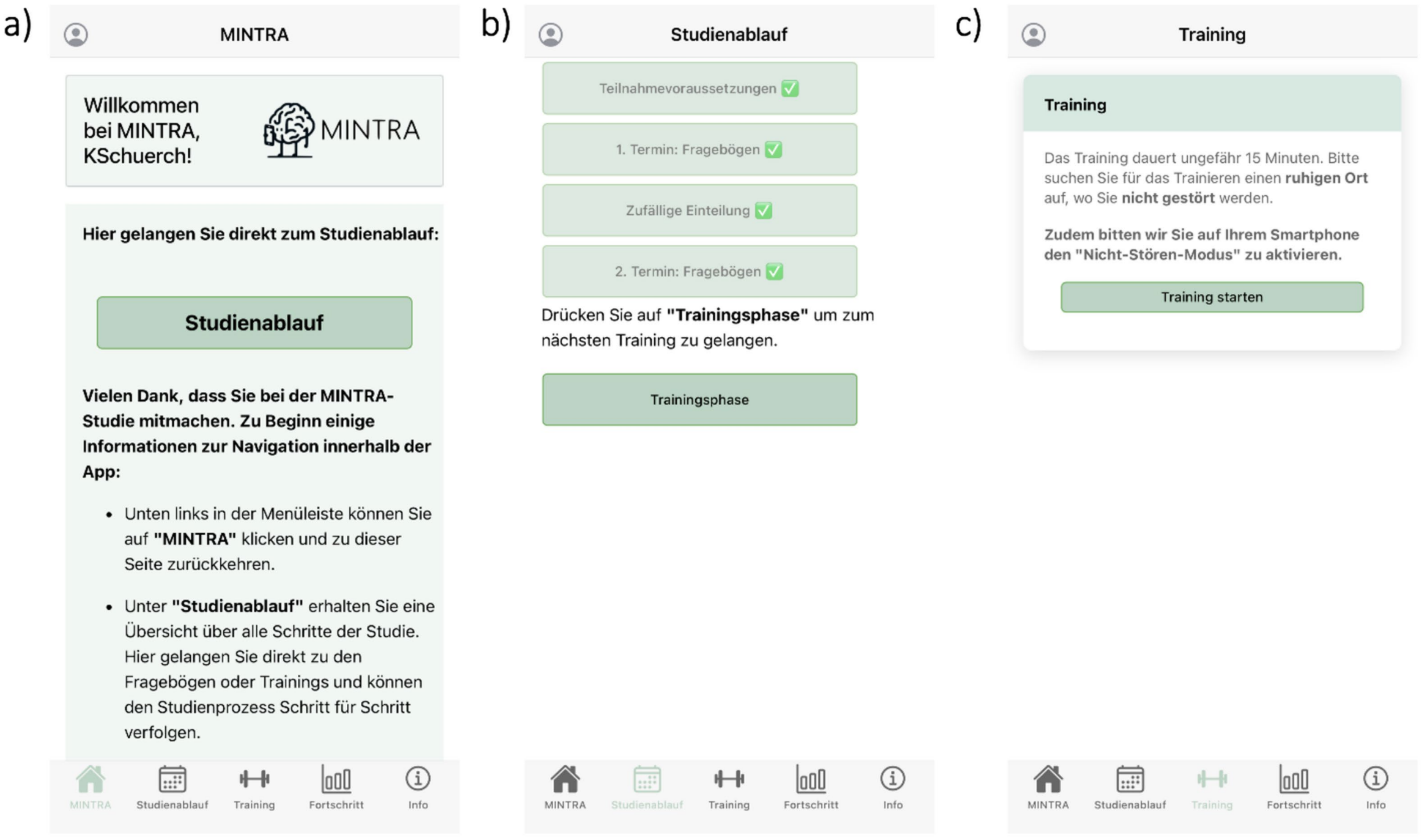
Illustrative screenshots of the MINTRA app interface in German. The app serves as the central platform for assessments, access to training, and progress tracking throughout the study. **(a)** Home screen welcoming the participant and providing navigation instructions. **(b)** Overview of the personal study timeline indicating completed steps and access to the current study timepoint. **(c)** Training start screen informing participants about session duration and instructions.

After providing written informed consent (Figure 1), participants will be invited to download the newly developed MINTRA app, which provides access to assessments and training and displays individual study timeline and training progress (see Figure 2). Participants undergo screening assessment during week 1 (T0), followed by the baseline assessment (T1), comprising self-report questionnaires and a structured telephone interview. Participants are then randomized to one of the two study groups (Alc-IT or Con-IT). Before the core training phase, a pre-training assessment with questionnaires (T2) is conducted. Over the subsequent two weeks, participants complete six short training sessions of their allocated version before a post-training assessment (T3, identical to T2) is conducted. One month later, the first follow-up assessment (T4) takes place after which participants complete six additional training sessions over a two-week period. The last follow-up assessment (T5) occurs two months after T4. Both follow-up assessments (T4 and T5) include questionnaires and telephone interviews. All questionnaires are completed within the app. In the EEG sub-study, pre- (T2) and post-training (T3) assessments will take place in the EEG lab and will be complemented by 64-channel EEG measurements during rest and during a Go/NoGo task (see 2.10). To promote adherence throughout the study, participants receive automated push notifications via the app as initial reminders. If adherence issues persist, participants are subsequently contacted by phone based on a structured contact scheme to encourage participation.

### 2.7 Study intervention and comparator

**Figure 3:**
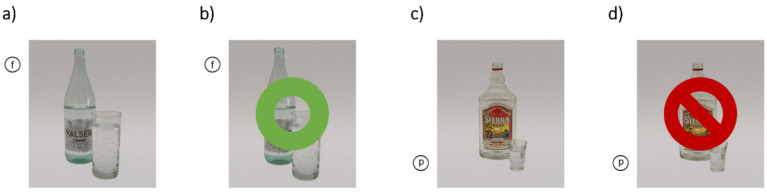
Illustrative screenshots of the study intervention. **(a)** A neutral picture with the letter cue “f” as a Go cue requiring a touch response **(b)** Correct response feedback (green circle) following a Go trial. **(c)** Alcohol-related picture (in this example: drink of choice = spirits) paired with the letter cue “p” as a NoGo cue where participants must withhold their response. **(d)** Incorrect response feedback: red crossed-out circle shown when a response was made following a NoGo trial.

Both training conditions are implemented as modified Go/NoGo tasks (Stein et al., 2023; Tschuemperlin et al., 2019), with identical visual layout, stimuli, and Go/NoGo cues. The conditions only differ in their respective probabilities of pairings between picture-type and NoGo-cues. Each session comprises 320 trials and takes about 10 to 15 min to complete. On each trial, a picture (alcohol-related or neutral) is presented in combination with a letter cue (“p” or “f”) appearing right outside one of the corners of the picture. The assignment of letters as Go or NoGo cues is counter-balanced across participants (see Figure 3). Neutral pictures are identical for all participants, whereas alcohol pictures are individually matched to each participant’s drink of choice (beer, wine, or spirits). Stimuli remain on screen for 1,500 ms. Participants are instructed to tap the screen as quickly and accurately as possible when a Go cue is shown and to withhold their response when a NoGo cue is shown. Visual feedback follows 500 ms after the (non)response (green circle = correct; red crossed-out circle = incorrect). Pictures and cue locations are randomized within each session.

- In the Alc-IT condition, alcohol-related pictures are solely combined with NoGo cues and the Go/NoGo ratio is 75/25.
- In the Con-IT condition, both picture types (alcohol-related, neutral) are paired with Go and NoGo cues equally often.

### 2.8 Risk management

Patients will be exposed to pictures of alcoholic beverages during the training. Even though this is not unusual, as patients tend to encounter (pictures of) alcohol in their everyday life as well, this might induce craving. Therefore, patients are asked to rate individual craving and stress levels after each session. In case of high values, emergency numbers will be made available in the Alc-IT app. It is important to note that in a prior RCT including the present alcohol-specific inhibition training with pictures of alcohol, no adverse events relating to the training were found (Stein et al., 2023; Tschuemperlin et al., 2019).

### 2.9 Outcome measures

#### 2.9.1 Demographics

Relevant demographics (e.g., age, gender, education) as well as relevant information about past AUD treatment or other mental health problems will be assessed.

#### 2.9.2 Screening

To evaluate eligibility, the following screening questionnaires will be used: The AUDIT (Babor et al., 2001), a screening questionnaire from the World Health Organization consists of 10 items about recent alcohol use, AUD symptoms, and alcohol-related problems. Self- rated symptoms of AUD will be assessed with the AUD-S (Knight et al., 2002), adapted to DSM-5 criteria as in Tschuemperlin et al. (2019). The DUDIT (Berman et al., 2005) is an 11-item self-administered screening instrument for drug-related problems, giving information on the level of drug intake.

#### 2.9.3 Primary outcome

Following the CONSORT guidelines’ extensions for pilot and feasibility trials (Eldridge et al., 2016), the feasibility hypothesis will be assessed by recruitment and completion rates (see H1), while treatment adherence and adverse events are collected as complementary data.

#### 2.9.4 Secondary outcomes

*Drinking behavior* is indicated by change in percentage of days abstinent and percentage of heavy drinking days. These indicators will help describe the preliminary effects of Alc-IT on drinking behavior and will be assessed using the timeline follow-back method (TLFB) at baseline (T1), 1-month (T4) and 3-month follow-up (T5). TLFB (Sobell and Sobell, 1992, 1995) is a procedure to aid recall of past drinking. Providing a relatively accurate estimate of one’s drinking, the TLFB is considered the gold-standard in assessing past drinking behavior. The TLFB will be administered by a telephone interview.

In the EEG sub-study, outcomes include *neurophysiological measures of inhibitory activity*, specifically changes in the N2 and/or P3 components of the event-related potential derived from NoGo trials (see 2.10 for task details and 2.11/2.12 for ERP computation). Additionally, the number of completed sessions, baseline neurophysiological parameters, impulsivity, or performance levels during training will be exploratorily assessed as moderators of the neurophysiological effects of Alc-IT. The topography of the P3 component, featured in hypothesis H3, is the main parameter of interest, while amplitude of the P3 component, as well as topography and amplitude of N2 will be assessed exploratorily.

#### 2.9.5 Additional outcomes

*Usability, user experience, and acceptability* will be additionally monitored to support the interpretation of the feasibility outcome (see objective 1): User Experience Questionnaire (UEQ; Schrepp, 2015); this 26-item self-report scale evaluates the user experience of interactive products on a 7-point Likert scale. System Usability Scale (SUS; Brooke, 2013); this 10-item self-report scale evaluates the usability of systems on a 5-point Likert scale. SUS is widely used to assess a broad range of technologies, offering a quick and reliable measure of perceived usability. Facets of Website Content Questionnaire (web-CLIC; Thielsch and Hirschfeld, 2019); this 12-item self-report measure originally evaluates the user experience and content of websites and will be tailored specifically for our app. Open-text and multiple-choice questions concerning drawbacks and strength of the app, technical problems encountered, suggestions for amelioration, intention to continue using the app, and place of using the app will complement this assessment.

#### 2.9.6 AUD, psychopathology, and related measures

Alcohol use is additionally assessed using eight adapted items from the Health and Daily Living Form (HDL, Moos et al., 1990), which has been validated in Swiss clinical samples (Moggi et al., 2010, 2007). Participants are also asked to indicate their personal drinking goals (e.g., total abstinence, controlled drinking, Moggi et al., 2010; Moggi et al., 2007), as these may change during treatment and in turn influence the post-treatment drinking outcome (Meyer et al., 2014). Alcohol craving is measured using the German version of the Obsessive-Compulsive Drinking Scale (OCDS-G, Mann and Ackermann, 2000). Additionally, three items rated on a Likert-scale assess the mean and maximum intensity and the frequency of craving over the past seven days (Moggi et al., 2007, 2010). Motivation to change is captured using the German version of the “Taking Steps” subscale from the SOCRATES (Miller and Tonnigan, 1996; Wetterling and Veltrup, 1997), alongside three items derived from motivational interviewing (Demmel, 2005; Miller and Rollnick, 2015), and a single item assessing self-efficacy regarding future alcohol reduction (Ludwig et al., 2013), which are rated on a Likert scale. Psychopathological symptoms are assessed with the 53-item Brief Symptom Checklist, German version (BSCL, Derogatis, 1993; Franke, 2000), yielding scores across nine symptom dimensions and three global indices. Subjective quality of life is measured using the Quality of Life-questionnaire (WHOQOL-BREF, Angermeyer et al., 2000). Emotion regulation is assessed with the 36-item Difficulties in Emotion Regulation Scale (DERS, Gratz and Roemer, 2004). The trait “impulsivity” will be assessed with the Skala Impulsives-Verhalten-8 (I-8, Kovaleva et al., 2012), a validated German short questionnaire to determine the multidimensional construct “impulsivity.” The German version of the ADHD Self Report Scale (ASRS-V1.1, Kessler et al., 2005; Lauth and Minsel, 2014) will be used to screen for ADHD related symptoms. In the EEG sub-study, the 20 items from the BIS (Behavioral Inhibition System) / BAS (Behavioral Approach System) questionnaire will additionally assess behavioral inhibition or approach tendencies (Carver and White, 1994). As in the big C-SURF cohort-study, we will use a slightly adapted version from the validated German version (Strobel et al., 2001).

### 2.10 Go/NoGo task

Inhibitory functions will be assessed experimentally with a cued Go-NoGo task (Batschelet et al., 2021; Stein et al., 2018) administered during EEG measurement on a computer in the EEG lab. During the task, alcohol-related and neutral pictures are presented in pseudorandomized order at a 1 Hz rate. Each picture is presented for 900 ms with the interstimulus interval jittered between 100 and 400 ms. Patients are asked to click as fast as possible whenever a new picture appears on screen (Go-trial), unless the same picture is repeated (NoGo-trial), resulting in both alcohol-related and neutral Go and NoGo-trials. Two pseudorandomized orders are established to ensure that unique sequences are presented at pre- and post-training assessments. Alcohol-related stimulus sets are tailored to the patient’s drink of choice (beer, wine, spirits). Stimulus sets consist of 20 alcohol-related and 20 neutral pictures, which were drawn from a validated and controlled stimulus set (Fey et al., 2017) in a way that ensured that (a) alcohol-related stimulus sets did not differ in arousal, craving, alcohol relatedness, valence, luminance, and visual complexity, and (b) alcohol and neutral stimulus sets did not differ in luminance and visual complexity (all *p*-values in ANOVA-based comparisons > 0.05). Each picture is presented 23 times during the task. With 800 Go trials (400 neutral, 400 alcohol-related) and 120 NoGo-trials (60 neutral, 60 alcohol-related), the task operates with Go-NoGo-ratio of 6.67, comprising of a total of 920 trials and lasts approximately 18 min. Given the different picture types (alcohol, neutral) and assessment time points (pre-, post-training), this task allows to compare error rates, reaction times and event-related potentials between picture types, time points and training groups and thus helps to investigate working mechanism of Alc-IT.

### 2.11 EEG recording and data reduction

Multichannel-EEG will be recorded in a sound-proof and electrically shielded chamber with a Nihon-Kohden Neurofax 1,100 system from 64 scalp electrodes placed according to the extended 10/20-system. All channels are online referenced against the mean of C3 and C4 and digitally sampled at 500 Hz. Electrode impedances will be kept below 20 kΩ, which is within the recommended range for active electrodes (Brain Products GmbH, 2022). On-line filters will be set at 0.016 Hz (high pass) and 150 Hz (low pass). Off-line, data will be preprocessed using BrainVision Analyzer (Version 2.0, Brain Products GmbH, Gilching, Germany). Preprocessing will include artifact correction via independent component analysis (to remove eye-movement and electrocardiac artifacts) and visual inspection (for remaining artifacts), interpolation of noisy channels, re-referencing to average reference and band-pass filtering. For the computation of event-related potentials (ERPs), clean data will be segmented into epochs from 500 ms before to 1,500 ms after stimulus onset. Individual ERPs will be computed separately for each picture type (alcohol, neutral), response type (Go, NoGo), and time point (pre- and post-training). To isolate inhibitory processes, difference waveforms (dERPs; NoGo minus Go) will be calculated for each condition.

### 2.12 Statistical analysis

#### 2.12.1 Objective 1 – feasibility of Alc-IT

Following the CONSORT guidelines’ extensions for pilot and feasibility trials (Eldridge et al., 2016), feasibility outcomes will be assessed primarily using data on recruitment and completion rates (indicating attrition), and additionally with treatment adherence, and adverse events. For these indicators, the parameters recommended in the CONSORT guidelines (such as means, data distributions, and percentages as well as narrative descriptions) will be reported. Failure to meet the above-defined indicators for promising feasibility will indicate that H1 cannot be confirmed. No inferential statistical testing will be performed for feasibility outcomes.

#### 2.12.2 Objective 2 – preliminary findings regarding the effectiveness of Alc-IT on drinking behavior

The present feasibility RCT is not designed to statistically test hypotheses regarding effectiveness outcomes but will instead provide preliminary data to estimate the effect sizes expected in a future full-scale RCT (Eldridge et al., 2016; Shanyinde et al., 2011; Thabane and Lancaster, 2019). In line with the CONSORT recommendations (Eldridge et al., 2016), preliminary effectiveness will be analyzed descriptively and using effect size indicators. Analyses will follow an intention-to-treat approach. Changes in drinking behavior over time will be analyzed with multilevel mixed-effects models for repeated measures, using baseline measures as the reference for estimating individual change trajectories. The most appropriate model structure, including relevant covariates (e.g., age, treatment setting, baseline severity, nicotine or cannabis use), will be selected based on model fit statistics. Estimated marginal means and standard errors will be reported. Cohen’s d will be calculated as an effect size estimate and presented alongside measures of uncertainty (Eldridge et al., 2016; Thabane et al., 2010). Sensitivity analyses stratified by setting and treatment received will complement the main analyses.

#### 2.12.3 Objective 3 – EEG sub-study: investigating the neurophysiological effects of iAlc-IT

Statistical comparisons of amplitude and topography of the N2 and P3 components (~150–850 ms) will be performed using the RAGU software package for whole-scalp randomization-based statistics, with exact time windows for N2 and P3 determined via microstate segmentation (Koenig et al., 2011, 2014). To test hypothesis H3, a three-way interaction between group (Alc-IT, Con-IT), picture type (alcohol, neutral), and time point (pre-, post-training) on the P3 topography will be examined by subjecting the difference ERPs to a topographical analysis of variance (TANOVA). Additional exploratory analyses will investigate whether neurophysiological effects are moderated by the number of completed sessions, baseline ERP characteristics, impulsivity scores, or training performance indices.

### 2.13 Data collection and management

Data will be collected through self-report questionnaires within the study app, Timeline Follow-Back interviews conducted by telephone, and EEG recordings for the EEG sub-study. Data collected through telephone interviews will be entered manually into the electronic system and securely stored at the study site with access restricted to authorized study personnel. The app automatically records usage metrics and questionnaire responses on a secure server hosted at the University of Bern. EEG data will be stored in compliance with institutional data protection policies, which is approved by the ethics committee of the canton of Bern. A separate list linking participant IDs to names is securely stored and restricted to authorized personnel. Data monitoring is performed regularly by an independent quality assurance representative who is not otherwise involved in the study. Anonymized data will be stored securely for at least 10 years after study completion in accordance with institutional and regulatory requirements.

## 3 Discussion

Computerized cognitive training interventions, such as Alc-IT, add a new component to traditional treatment approaches for AUD. These interventions guide patients through the numerous repetitions required to modulate automatized, partly subcortically anchored behaviors. As a result, they offer a cost-effective way to enhance therapy and improve treatment outcome (Batschelet et al., 2020; Stein et al., 2023; Wiers, 2018). Adapting these interventions to flexible app-based formats and addressing a younger population could further enhance their potential, provided such an application is feasible, acceptable, and effective – objectives directly addressed in the present study. Integrating Alc-IT into outpatient or (online) counseling settings may provide an innovative early intervention component aimed at disrupting the progression toward habitual or even compulsive alcohol use in its early stages. This aligns with public health goals of preventing long-term impairments in psychosocial functioning and reducing the risk of subsequent mental health disorders.

Especially during adolescence and young adulthood, neurodevelopmental changes create a temporary imbalance between subcortical reward systems and prefrontal control networks (Casey et al., 2008; Casey and Jones, 2010; Galvan et al., 2006). This developmental asynchrony leads to heightened reward sensitivity and reduced top-down regulation, contributing to increased risk-taking and experimentation with substances during this phase (Casey and Jones, 2010; Pieters et al., 2014; Wiers et al., 2010). Neuroscientific models of AUD describe a similar imbalance, where strong appetitive drives and weak inhibitory control jointly promote maladaptive, cue-driven behavior (Volkow and Baler, 2014; Wiers et al., 2007). From this perspective, interventions that strengthen inhibitory control may be particularly beneficial during adolescence, when inhibitory processes are still developing.

A feasibility RCT was chosen because Alc-IT has not yet been tested as an app-based version in an outpatient or (online) counseling setting. This design is appropriate to evaluate whether the implementation of Alc-IT in these treatment settings is practicable before conducting larger-scale confirmatory studies. One potential risk is that changes in patient characteristics at participating treatment centers – such as shifts in substance use patterns among young patients – could make recruitment more difficult. Additionally, app-based interventions frequently encounter challenges related to user adherence and attrition (Schwartz et al., 2024). Identifying critical moments for adherence can help improve procedures for broader implementation. Given these challenges, a feasibility RCT allows for early identification of difficulties related to recruitment, usability, and attrition. Gathering reliable experience and – if necessary – testing the capacity of mitigation measures to counteract these difficulties is thus an important aim of this study. Such measures could, for example, include refining the app design, optimizing motivational components, or tailoring communication and support strategies to enhance adherence and user engagement.

Considering the scientific implications, the proposed project seeks to not only inform future full-scale RCTs but also deepen our understanding of the neurophysiological basis of AUD and its modifiability through training interventions. This is even more important, as research on mechanisms of change in Alc-IT is scarce and has never been done in a young clinical sample. Due to shared etiological aspects and shared change mechanisms, the expected results might in part also be transferable to other substance use disorders or even behavioral addictions, for which such an intervention might be adapted.

While this feasibility study includes additional psychological and behavioral measures, its primary aim is to evaluate the implementation of app-based Alc-IT. Future studies should extend outcome assessment beyond drinking behavior to include indicators such as general well-being and self-efficacy. Such measures could be critical for understanding the broader benefits of Alc-IT. In addition, incorporating complementary neuropsychological tasks could help determine whether training effects generalize beyond alcohol-specific contexts or remain cue-specific. Given the feasibility focus and the available resources of the current study, these additional assessments were not included at this stage. However, insights gained from this study will inform the design and prioritization of such extensions in future full-scale studies.

Taken together, the planned feasibility RCT evaluates the feasibility of app-based Alc-IT in younger outpatients, aiming to identify and address challenges related to recruitment and adherence. Additionally, it aims to inform future trials by enhancing our understanding of change mechanisms in AUD by tracking neurophysiological effects of app-based inhibition training.

## Footnotes

1 Note that these expectations are based on the last available yearly admission rates to the five treatment centers (combined *N* = 4,670), the mean proportion of patients being aged 14–27 years (24%; *N* = 1,120) and additionally meeting the inclusion/exclusion criteria (45%, *N* = 504), leading to *N* = 42 patients approachable per month. In combination with the expected completion rates (see H1 in section 1.1), the resulting sample size at follow-up is *N* = 90.
